# Suppressing VEGF-A/VEGFR-2 Signaling Contributes to the Anti-Angiogenic Effects of PPE8, a Novel Naphthoquinone-Based Compound

**DOI:** 10.3390/cells11132114

**Published:** 2022-07-05

**Authors:** Ming-Jen Hsu, Han-Kun Chen, Jin-Cherng Lien, Yu-Han Huang, Shiu-Wen Huang

**Affiliations:** 1 Graduate Institute of Medical Sciences, College of Medicine, Taipei Medical University, Taipei 11031, Taiwan; aspirin@tmu.edu.tw; 2 Department of Pharmacology, School of Medicine, College of Medicine, Taipei Medical University, Taipei 11031, Taiwan; 3 Cell Physiology and Molecular Image Research Center, Wan Fang Hospital, Taipei Medical University, Taipei 11031, Taiwan; 4 Department of General Surgery, Chi Mei Medical Center, Tainan 71067, Taiwan; d930405@mail.chimei.org.tw; 5 School of Pharmacy, China Medical University, Taichung 40402, Taiwan; jclien@mail.cmu.edu.tw; 6 Department of Medical Research, Hospital of China Medical University, Taichung 40402, Taiwan; 7 Division of Genetics and Genomics, Department of Pediatrics, Boston Children’s Hospital and Harvard Medical School, Boston, MA 02115, USA; yu-han.huang@childrens.harvard.edu; 8 The Manton Center for Orphan Disease Research, Boston Children’s Hospital, Boston, MA 02115, USA; 9 Department of Medical Research, Taipei Medical University Hospital, Taipei 11031, Taiwan; 10 Research Center of Thoracic Medicine, Taipei Medical University Hospital, Taipei 11031, Taiwan

**Keywords:** angiogenesis, human umbilical vein endothelial cells (HUVECs), naphthoquinones, vascular endothelial growth factor (VEGF)

## Abstract

Natural naphthoquinones and their derivatives exhibit a broad spectrum of pharmacological activities and have thus attracted much attention in modern drug discovery. However, it remains unclear whether naphthoquinones are potential drug candidates for anti-angiogenic agents. The aim of this study was to evaluate the anti-angiogenic properties of a novel naphthoquinone derivative, PPE8, and explore its underlying mechanisms. Determined by various assays including BrdU, migration, invasion, and tube formation analyses, PPE8 treatment resulted in the reduction of VEGF-A-induced proliferation, migration, and invasion, as well as tube formation in human umbilical vein endothelial cells (HUVECs). We also used an aorta ring sprouting assay, Matrigel plug assay, and immunoblotting analysis to examine PPE8’s ex vivo and in vivo anti-angiogenic activities and its actions on VEGF-A signaling. It has been revealed that PPE8 inhibited VEGF-A-induced micro vessel sprouting and was capable of suppressing angiogenesis in in vivo models. In addition, PPE8 inhibited VEGF receptor (VEGFR)-2, Src, FAK, ERK1/2, or AKT phosphorylation in HUVECs exposed to VEGF-A, and it also showed significant decline in xenograft tumor growth in vivo. Taken together, these observations indicated that PPE8 may target VEGF-A–VEGFR-2 signaling to reduce angiogenesis. It also supports the role of PPE8 as a potential drug candidate for the development of therapeutic agents in the treatment of angiogenesis-related diseases including cancer.

## 1. Introduction

Sprouting angiogenesis is necessary for embryonic development, reproduction, and wound repair. Besides these physiological processes, neovascularization also plays a pivotal role in various human diseases including psoriasis, atherosclerotic lesion formation, and ophthalmological diseases, as well as tumor metastasis and progression [1]. Tumor neovascularization contributes not only to tumor growth and the metastatic spread of tumor cells, but also tumor immunosuppression [2,3]. In addition, the combination of immune checkpoint inhibitors and anti-angiogenic therapy has recently shown its beneficial effects in several types of malignancy [4,5,6,7]. According to the report by the World Health Organization (WHO) on annual world health statistics, cancer remains a major cause of death and the major life burden worldwide. Therefore, it is crucial to develop novel therapeutic agents targeting angiogenesis for the future treatment of these angiogenesis-related diseases and cancer intervention [8]. Angiogenesis could be initiated by pro-angiogenic factors of cytokines including VEGF-A, platelet-derived growth factor (PDGF), fibroblast growth factor (bFGF) [9], and IL-6 [10]. Among these factors, the founding member of VEGF family, VEGF-A, is recognized as the main mediator of angiogenesis. VEGF-A binds to VEGF receptor (VEGFR)-2 and activates its downstream pathways such as focal adhesion kinase (FAK), Src, extracellular-signal-regulated kinase (ERK)1/2, and Akt [11]. This leads to the promotion of cell proliferation, migration, invasion, and tube formation of vascular endothelial cells, the critical steps of angiogenesis [12,13]. Therefore, targeting VEGF-A-VEGFR-2 signaling represents a rational strategy for limiting angiogenesis and tumor metastasis. 

Bevacizumab (Avastin^®^), the first neutralizing monoclonal anti-VEGF-A antibody approved by the United States Food and Drug Administration (U.S. FDA), is the most widely used anti-angiogenic drug [14]. Ramucirumab (Cyramzar^®^) [15] is another monoclonal antibody targeting VEGFR-2 used for limiting angiogenesis. In addition to neutralizing antibodies, strategies aimed to alleviate tumor angiogenesis also include antisense oligonucleotides that target VEGF [16], soluble decoy VEGF receptors [17], and small molecule inhibitors that target VEGF-A–VEGFR-2 signaling [18]. To date, small molecule inhibitors such as sunitinib (Sutent^®^), axitinib (Inlyta^®^), sorafenib (Nexavar^®^), regorafenib (Stivarga^®^), pazopanib (Votrient^®^), and vandetanib (Caprelsa^®^) have already been approved by U.S. FDA or the European Medicines Agency (EMEA) for the treatment of cancer in the adjuvant setting [19]. 

Natural products and their derivatives play critical roles in modern drug discovery and have been recognized as an important source of novel therapeutic agents. Naturally occurring naphthoquinones are the secondary metabolites widely distributed in higher plants, fungi, and microorganisms. There is increasing evidence that naphthoquinone derivatives display broad-spectrum pharmacological activities including anti-viral, anti-bacterial, and anti-inflammatory, as well as anti-tumor activities [20,21,22,23,24]. The naphthoquinone derivative, vitamin K, has been used in the treatment of hemorrhagic diseases [25]. Several naphthoquinone-based compounds including doxorubicin, idarubicin, and mitoxantrone have been already approved as chemotherapeutic agents against certain types of cancer [26,27]. In addition, lapachone, lapachol, or napabucasin with naphthoquinone pharmacophore are currently in clinical trials for cancer treatment [28]. It appears that additional naphthoquinones may exhibit pharmacological properties capable of clinical application. We previously synthesized two novel small molecules with 1, 4 naphthoquinone pharmacophore, namely PPE and PPE8. We demonstrated that PPE8 exhibits anti-tumor effects [29]. Recent studies showed that 1, 4 naphthoquinone derivatives are also effective in suppressing angiogenesis, although the underlying mechanisms remain to be clarified [30]. Given their potential as drug candidates for the development of novel anti-angiogenic agents, we aim to evaluate the anti-angiogenic properties of PPE8 as well as to investigate the mechanisms underlying PPE8’s anti-angiogenic actions in vascular endothelial cells.

## 2. Materials and Methods

### 2.1. Reagents

Recombinant VEGF-A was from PeproTech (Rocky Hill, NJ, USA). DMEM, fetal bovine serum (FBS), TrypLE™, Medium 199 (M199), and all cell culture reagents were from Invitrogen (Carlsbad, CA, USA). Antibodies against VEGFR-2, anti-phospho-VEGFR-2 (Y1175), Akt and anti-phospho-Akt (S473), ERK1/2 and anti-phospho-ERK1/2 (T202/Y204), FAK, anti-phospho-FAK (Y397), Src and anti-phospho-Src phosphorylated (Y416), were from Cell Signaling (Danvers, MA, USA). Antibody against α-tubulin and anti-mouse and anti-rabbit IgG-conjugated horseradish peroxidase antibodies were from GeneTex Inc (Irvine, CA, USA). All materials for immunoblotting were from Bio-Rad (Hercules, CA, USA). BD Matrigel^TM^ basement membrane matrix was from Becton Dickinson (Mountain View, CA, USA). The immobilon Western chemiluminescence HRP substrate was from Millipore (Billerica, MA, USA). Cell Proliferation ELISA, BrdU assay kit was from Roche (Indianapolis, IN, USA). Toluidine blue O, 3-[4,5-dimethylthiazol-2-yl]-2, 5-diphenyltetrazolium bromide (MTT) and all other chemicals were obtained from Sigma-Aldrich (St Louis, MO, USA). 

### 2.2. Synthesis of PPE8

PPE8, a naphthoquinone-based compound, was synthesized as described previously [29]. 

### 2.3. Cell Culture

The B16F10 melanoma cell line, MDA-MB-231 breast cancer cell line, primary human umbilical vein endothelial cells (HUVECs), and Hs68 human foreskin fibroblast cell line were purchased from the Bioresource Collection and Research Center (Hsinchu, Taiwan). The HaCat human immortalized keratinocyte cell line was from DKFZ (Heidelberg, Germany). B16F10, MDA-MB-231, HaCat, and Hs68 cells were maintained in 10%-FBS-containing DMEM medium in the presence of 100 U/mL penicillin G, 100 μg/mL streptomycin, and 0.25 μg/mL amphotericin B in a humidified 37 °C incubator. HUVECs were cultured in 10%-FBS-containing M199 medium in the presence of endothelial cell growth supplement (ECGS) (Millipore, Billerica, MA, USA), 100 U/mL penicillin G, 100 μg/mL streptomycin, 0.25 μg/mL amphotericin B (Biological Industries, Cromwell, CT, USA), 20 mM HEPES, and 5 U/mL heparin, in a humidified 37 °C incubator. 

### 2.4. MTT Assay 

A colorimetric MTT assay was used to determine cell viability as described previously [31]. 

### 2.5. Cell Proliferation Assay

HUVECs seeded in 96-well tissue culture plates (2 × 10^4^ cells/well) were starved in 2%-FBS-containing M199 medium without endothelial cell growth supplements for 18 h. After 30 min treatment with PPE8 at indicated concentrations, cells were stimulated with VEGF-A (25 ng/mL) for another 24 h. A Cell Proliferation ELISA, BrdU assay kit (Millipore, Billerica, MA, USA) was used to examine the extent of cell proliferation based on the colorimetric detection of the incorporation of BrdU as per the manufacturer’s instructions.

### 2.6. Cell Migration Assay 

After reaching confluence, HUVECs seeded in 0.1% gelatin-coated 12-well tissue culture plates were starved in M199 medium containing 2% FBS in the absence of ECGS (Millipore, Billerica, MA, USA) for 18 h. The endothelial monolayer was wounded by scratching with tips after starvation. Cells were treated with PPE8 at indicated concentrations in the presence or absence of VEGF-A (25 ng/mL) for another 24 h. Cells were fixed for 30 min with paraformaldehyde (4%) and stained with toluidine blue O (0.5%). An *OLYMPUS* Biological Microscope digital camera (Yuan Li Instrument Co., Taipei, Taiwan) was used to take the images at 40× magnification. To evaluate the gap closure rate, an Image J program (http://rsbweb.nih.gov/ij/index.html (accessed on 13 June 2022)) was employed to compare the sizes of the scratch area as a percentage of the values obtained with their respective controls at the beginning of the experiment (time 0).

### 2.7. Cell Invasion Assay

To perform the cell invasion assays, Transwell plates (Corning, NY, USA) were used as previously described [31]. Briefly, the bottom face of the insert membrane was covered with 0.2% gelatin. HUVECs in 200 µL 2%-FBS-containing M199 medium (10^4^ cells/well) in the presence or absence of PPE8 were seeded in the top chambers. The bottom chambers were filled with M199 medium containing 2% FBS with or without 25 ng/mL VEGF-A. After 18 h treatment, non-invaded cells were scraped with a cotton swab. Invaded cells (on the bottom side of the insert membrane) were fixed for 30 min with paraformaldehyde (4%) and stained with toluidine blue O (0.5%). An inverted contrast-phase light microscope (Nikon, Japan) was used to photograph the invaded cells at 40× magnification. The extent of cell invasion was determined by counting the invaded cells in three random fields.

### 2.8. Tube Formation Assay

Matrigel was polymerized for 30 min at 37 °C. HUVECs suspended in M199 medium containing 2% FBS in the presence or absence of 25 ng/mL VEGF-A were then seeded onto the Matrigel. After seeding, the cells were treated with or without PPE8 at indicated concentrations for 18 h. An inverted contrast-phase light microscope (Nikon, Japan) was used to photograph the formed tubes at 40× magnification. To quantify the formed tube network, the number of tube sprout arches was counted in three random fields.

### 2.9. Animals

All animal care and experimental protocols were approved by the Taipei Medical University Laboratory Animal Care and Use Committee (Permit Number: LAC-2019-0399) and complied with the recommendations in the Guide for the Care and Use of Laboratory Animals of the National Institutes of Health (NIH publication No. 85-23, revised 1996). Animal studies are reported in compliance with the ARRIVE guidelines [32].

### 2.10. Rat Aortic Ring Sprouting Assay

Six male Sprague-Dawley rats (8- to 10-week-old) from the National Laboratory Animal Center (Taipei, Taiwan) were used for the aortic ring sprouting assay as previously described [31]. Rats were sacrificed using CO_2_ asphyxiation to dissect the aortic arches. The surrounding fibro-adipose tissues were removed. The aortas were rinsed thoroughly with M199 medium and cut into approximately 1 mm ring segments. In each experiment, the aortic rings obtained from one rat were utilized for different treatment groups. The aortic rings were immersed in Matrigel and treated with VEGF-A (25 ng/mL) in the presence or absence of PPE8. The treated aortic rings were placed in a humidified 37 °C incubator and the medium was changed every 3 days. An inverted contrast-phase light microscope (Nikon, Japan) was used to photograph the growing sprouts of endothelial cells at 40× magnification on day 7. The sprouting area was determined by an observer who was unaware of the treatment group. Image-Pro Plus software (Media Cybernetics, Inc., Rockville, MD, USA) (Image-Pro Plus) was employed to quantify the sprouting area.

### 2.11. In Vivo Matrigel Plug Angiogenesis Assay

To determine PPE8’s in vivo anti-angiogenic effects, a Matrigel plug angiogenesis assay as previously described [31] was used. Six male nude_nu/nu_ mice (3- to 5-week-old) with a body weight of about 20 g were purchased from the National Laboratory Animal Center (Taipei, Taiwan). All mice were allocated randomly to an individually ventilated cage (IVC) by vivarium staff upon transfer from the National Laboratory Animal Center (Taipei, Taiwan) into the clean specific-pathogen-free (SPF) rooms and were acclimatized in the animal housing room for 7 days before starting experiments. The cage floor was covered with *Bed* O’Cobs animal bedding (The Andersons, Maumee, OH, USA). All the mice (3 mice per cage) were maintained on standard chow and autoclaved water. 

Mice were anesthetized with intraperitoneal Zoletil (15 mg/100g) plus Xylazine (0.23 mg/100g). 

Once anesthesia was induced, a heparin (20 U)-containing aliquot of Matrigel (250 μL) was mixed with MDA-MB-231 human breast cancer cells suspended in PBS in a volume of 150 μL and subcutaneously injected into the right flank of each nude_nu/nu_ mouse (tumor-cell-induced angiogenesis model). In another set of experiments, a VEGF-A (100 ng/mL)-containing aliquot of Matrigel (500 μL) in the presence of heparin (20 U) was subcutaneously injected into the right flank of each C57BL/6 mouse (VEGF-A-induced angiogenesis model). Mice were randomized to either the control group or the PPE8-treated group. PPE8 was administrated intraperitoneally once daily for 10 (MDAMB231-cell-induced angiogenesis model) or 7 (VEGF-A-induced angiogenesis model) days. Mice were sacrificed by carbon dioxide asphyxiation at the end of treatment. Matrigel plugs were removed, and the surrounding tissues were trimmed. Hemoglobin level was used as a parameter for vasculature for Matrigel angiogenesis quantification. Isolated Matrigel plugs were sonicated in PBS, allowing blood components to be dissolved in the solution. To determine the hemoglobin levels of the derived supernatant, a Drabkin’s reagent kit (Sigma-Aldrich) was employed as per the manufacturer’s instructions.

### 2.12. Mouse Xenograft Model

To determine PPE8’s anti-tumor effects in vivo, a xenograft model with C57BL/6 mice was used as previously described [33]. Male C57BL/6 mice (4-week-old) were purchased from the National Laboratory Animal Center (Taipei, Taiwan). All the mice (5 mice per cage), maintained on standard chow and autoclaved water, were housed in clean conventional animal housing rooms in the Laboratory Animal Center of Taipei Medical University. All mice were acclimatized in the animal housing room for 7 days before starting experiments. After acclimatization, mice (about 5- to 6-week-old) with a body weight of about 20 g were used for the xenograft model. B16F10 melanoma cells (4 × 10^6^ cells) suspended in PBS in a volume of 200 μL were subcutaneously injected into the flank of each mouse. Once the tumor reached approximately 100 mm^3^, mice were randomized to either the control group (6 mice) or the PPE8-treated group (6 mice). PPE8 was administrated intraperitoneally once daily for 30 days. Tumors were measured every 3 days using a digital caliper. The formula *V* (mm_3_) = [*ab*_2_] × 0.52, where *a* is the length and *b* is the width of the tumor [34], was used to calculate tumor volume. The body weights of the mice were examined every 3 days during the 30 day-treatment of PPE8 or vehicle. Mice were sacrificed by CO_2_ asphyxiation at the end of treatment and tumors were removed and weighed. 

### 2.13. Immunohistochemically Analysis

The proliferative cells were determined on the cryosections of B16F10 xenograft tumors using a rabbit anti-Ki67 antibody (Novus Biologicals, Littleton, CO, USA) and goat anti-rabbit antibody conjugated with peroxidase (The Jackson Laboratory, Sacramento, CA, USA). The proliferative cells (Ki67^+^ area) were visualized using stable diaminobenzidine. Images were obtained from each section at ×100 magnification. The area of Ki67-stained proliferative cells was examined as previously described [34].

### 2.14. Immunoblotting

Cells were harvested in a lysis buffer containing 2 mM PMSF, 0.05 mM pepstatin A, 0.2 mM leupeptin, 0.5% NP-40, 10 mM Tris (pH 7.0), and 140 mM NaCl. Equal amounts of protein samples were subjected to SDS-PAGE and transferred onto an NC membrane (Pall Corporation, Washington, NY, USA). The NC membrane was incubated for 1 h with blocking buffer containing 5% non-fat milk. Proteins were recognized after incubations for 2 h with specific primary antibodies, followed by incubation with secondary antibodies conjugated with horseradish peroxidase for another 1 h. The immobilon Western chemiluminescence HRP substrate (Billerica, MA, USA) was employed to detect immunoreactivity as per the manufacturer’s instructions. A computing densitometer with the scientific imaging system (Biospectrum AC System, UVP) was used to quantify the intensity of immunoblot bends. 

### 2.15. Data and Statistical Analysis 

In each experiment, we used the same cell to determine PPE8’s effects versus the relative control. Therefore, formal randomization was not used. We also performed normalization to compare the differences after the treatment to reveal relevant trends and to control for unwanted sources of variation. The results shown in this study are expressed as mean ± SEM; n ≥ 5, where “n” refers to independent values, and not replicates. The group data, which have a minimum of n = 5 independent samples per group, were subjected to statistical analysis. We used SigmaPlot 14.5 (Build 10.0.0.54; Systat Software, San Jose, CA, USA; SigmaPlot) to perform statistical analysis. Statistical comparisons between two groups were determined by the Mann–Whitney test for non-parametric analysis or unpaired Student’s t-test for parametric analysis. Statistical comparisons among more than two groups were evaluated by Kruskal–Wallis test followed by Dunn’s multiple comparisons for non-parametric analysis or one-way ANOVA with Tukey’s post hoc test for parametric analysis. A *p* value smaller than 0.05 was defined as statistically significant. The immunoblotting data were subjected to non-parametric statistical analysis.

## 3. Results

### 3.1. PPE8, a Novel Naphthoquinone-Based Compound Reduced Cell Proliferation, Migration, and Invasion of VEGF-A-Stimulated HUVECs

**Figure 1:**
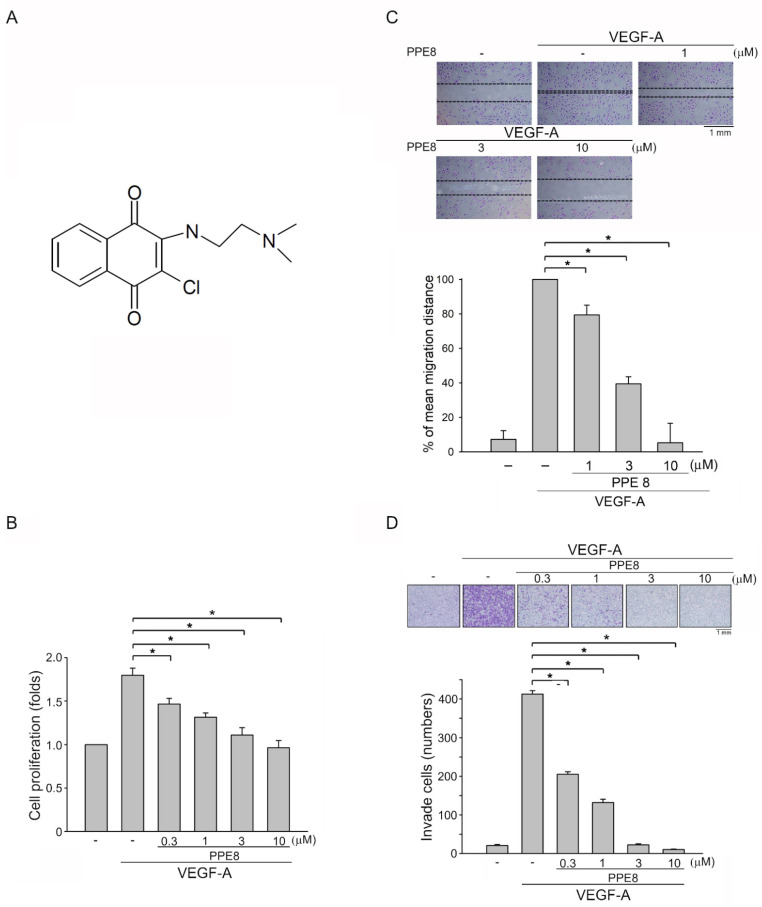
PPE8 suppressed HUVEC migration, invasion, and proliferation after VEGF-A exposure. (**A**) Chemical structure of PPE8. (**B**) After starvation for 18 h with M199 medium containing 2% FBS in the absence of ECGS, HUVECs were pre-treated with PPE8 for 30 min. Cells were then stimulated with VEGF-A (25 ng/mL) for another 24 h. A BrdU-based cell proliferation assay was used to determine cell proliferation. Each column represents the mean ± S.E.M. of six independent experiments performed in duplicate. * *p* < 0.05, compared with the group treated with VEGF-A alone; Kruskal–Wallis test. (**C**) HUVECs were starved as described in (**B**). HUVECs were scratched and treated with PPE8 or vehicle for 30 min, followed by the stimulation with VEGF-A for another 24 h. The migration distance was determined. Each column represents the mean ± S.E.M. of six independent experiments * *p* < 0.05, compared with the group treated with VEGF-A alone; one-way ANOVA, with Tukey’s post hoc test. (**D**) After starvation as described in (**B**), PPE8’s effects on VEGF-A-induced cell invasion was determined as described in the “Materials and Methods” section. Invaded HUVECs were stained and quantified. Each column represents the mean ± S.E.M. of six independent experiments. * *p* < 0.05, compared with the group treated with VEGF-A alone; one-way ANOVA, with Tukey’s post hoc test.

Cell migration, invasion, and proliferation, as well as the tube formation of vascular endothelial cells, are critical angiogenic processes [9]. To determine whether PPE (Figure S1A) and PPE8 (Figure 1A), two novel naphthoquinone derivatives, exhibit anti-angiogenic properties, a BrdU incorporation assay was employed to determine their effects on HUVEC proliferation after VEGF-A exposure. After 18 h starvation with 2%-FBS-containing M199 medium, HUVECs were stimulated by VEGF-A (25 ng/mL) with or without these compounds for another 24 h. As compared with PPE, PPE8 at 10 μM exhibits higher inhibitory effects on cell proliferation in VEGF-A-stimulated HUVECs (Figure S1B). We thus selected PPE8 in the following experiments to investigate its anti-angiogenic actions. PPE8 inhibits VEGF-A-stimulated HUVEC proliferation in a concentration-dependent manner, with an IC50 of approximately 0.6 μM (Figure 1B). A cell migration assay was used to evaluate whether PPE8 affects HUVEC motility after VEGF-A exposure. PPE8 was shown to significantly suppress VEGF-A-induced cell migration (Figure 1C). Results derived from the cell invasion assay also show that PPE8 is capable of reducing VEGF-A-induced cell invasion (Figure 1D).

### 3.2. PPE8 Attenuated VEGF-A-elicited Tubular Formation of HUVECs and Microvessel Sprouting

**Figure 2:**
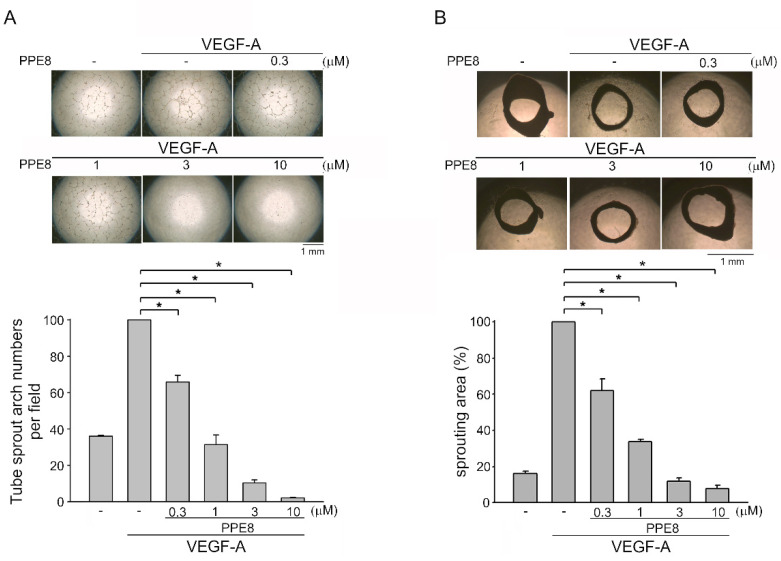
PPE8 suppressed tube formation of HUVECs in vitro and aorta ring sprouting ex vivo after VEGF-A exposure. (**A**) PPE8’s effects on VEGF-A-induced tubular formation of HUVECs were determined as described in the “Materials and Methods” section. Formed connected capillary-like tubes and mesh-like structures after 18 h exposure to VEGF-A were photographed under phase-contrast microscopy. The sprout arch numbers were counted. Each column represents the mean ± S.E.M. of six independent experiments. * *p* < 0.05, compared with the group treated with VEGF-A alone; one-way ANOVA, with Tukey’s post hoc test. (**B**) Rat aortic rings placed in Matrigel were treated with VEGF-A (25 ng/mL) plus vehicle or PPE8. At the end of the experiment, the percentage of sprouting microvessel area was evaluated. Each column represents the mean ± S.E.M. of six independent experiments. * *p* < 0.05, compared with the group treated with VEGF-A alone; one-way ANOVA, with Tukey’s post hoc test.

We evaluated whether the tubular formation of HUVECs after VEGF-A exposure is altered in the presence of PPE8. HUVECs seeded on Matrigel with or without PPE8 (0.3–10 μM) were treated with VEGF-A (25 ng/mL) for 24 h. Cells became elongated, formed connected capillary-like tubes, and created a mesh-like structure after VEGF-A exposure for 24 h. PPE8 treatment, however, resulted in impairment of the capillary-like network formation in response to VEGF-A (Figure 2A). We used an ex vivo rat aortic ring sprouting assay to further examine PPE8’s anti-angiogenic effects. As shown in Figure 2B, the sprouting microvessels were significantly increased and a complex network was formed around the aortic rings after VEGF-A exposure. However, this effect was significantly reduced by PPE8 (0.3-10 μM) (Figure 2B). Together these observations suggest that PPE8 may impair VEGF-A-induced angiogenesis by reducing migration, invasion, and proliferation, as well as the tube formation of vascular endothelial cells.

### 3.3. PPE8 Attenuated VEGF-A- or Tumor-Cell-Induced Angiogenesis In Vivo 

**Figure 3:**
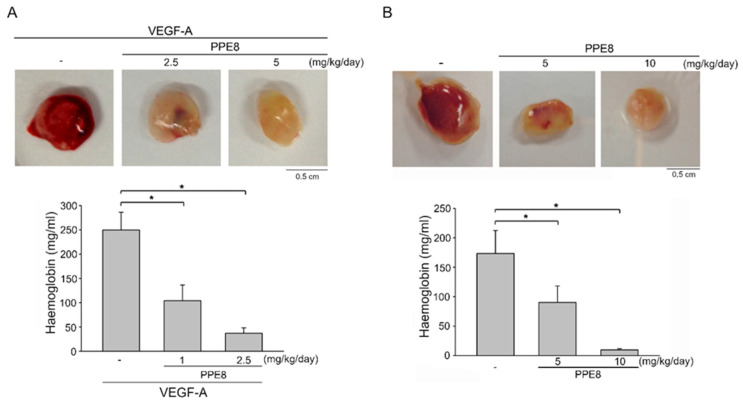
PPE8 reduced VEGF-A- or tumor-cell-elicited angiogenesis (**A**) PPE8’s in vivo effects on VEGF-A-induced angiogenesis were determined using a Matrigel plug assay as described in the “Materials and Methods” section. Matrigel plugs removed from the vehicle- or PPE8-treated mice are shown in the upper panel of the chart. Hemoglobin levels in the Matrigel plugs are shown in the bottom panel of the chart. Each column represents the mean ± S.E.M. of six plugs. * *p* < 0.05, compared with the group treated with VEGF-A alone; one-way ANOVA, with Tukey’s post hoc test. (**B**) PPE8’s in vivo effects on MDA-MB-231-breast-cancer-cell-induced angiogenesis was determined using a tumor-cell-induced angiogenesis Matrigel plug assay as described in the “Materials and Methods” section. Matrigel plugs removed from the vehicle- or PPE8-treated mice are shown in the upper panel of the chart. Hemoglobin levels in the Matrigel plugs are shown in the bottom panel of the chart. Each column represents the mean ± S.E.M. of six plugs. * *p* < 0.05, compared with the group treated with VEGF-A alone; one-way ANOVA, with Tukey’s post hoc test.

We next used a Matrigel plug angiogenesis assay to evaluate whether PPE8 is effective in reducing VEGF-A- or tumor-cell-elicited angiogenesis in vivo. Subcutaneously-implanted Matrigel plugs with VEGF-A (100 ng/mL) markedly induced microvessel formation within 7 days (Figure 3A). However, the plugs removed from the PPE8-treated mice showed pale color when compared with those from vehicle-treated mice, indicating that treatment with PPE8 reduced VEGF-A-induced neovascularization (Figure 3A, upper panel). We examined the hemoglobin content of the plugs to quantify the angiogenic response. As shown in Figure 3A, intraperitoneal administration of PPE8 led to a significant reduction in VEGF-A-induced angiogenesis (Figure 3A, bottom panel). We also used a tumor-cell-induced angiogenesis Matrigel plug assay to access PPE8’s anti-angiogenic effects in vivo. Matrigel mixed with MDA-MB-231 breast cancer cells was implanted subcutaneously into the flanks of mice. After 10-days treatment with PPE8 (5 or 10 mg/kg/day) or vehicle, MDA-MB-231 cells markedly induced neovascularization in the Matrigel plugs removed from the vehicle-treated mice (Figure 3B, upper panel). This effect was significantly reduced by PPE8 treatment (Figure 3B, upper panel). The angiogenic response was quantified by evaluating the hemoglobin content of the plugs. As compared with the vehicle-treated control group, PPE8 significantly attenuated MDA-MB-231-breast-cancer-cell-induced angiogenesis in vivo (Figure 3B, bottom panel). We also used a tail bleeding time analysis, a commonly used test to analyze hemostasis, to determine PPE8’s in vivo effects on the risk of hemorrhage or hemostasis. As shown in Figure S2, PPE8 was without effects on tail bleeding time. Together these observations suggest that the systemic administration of PPE8 is capable of reducing VEGF-A- or tumor-cell-induced angiogenesis in vivo.

### 3.4. PPE8 Reduced Endothelial VEGF-A-VEGFR-2 Signaling

**Figure 4:**
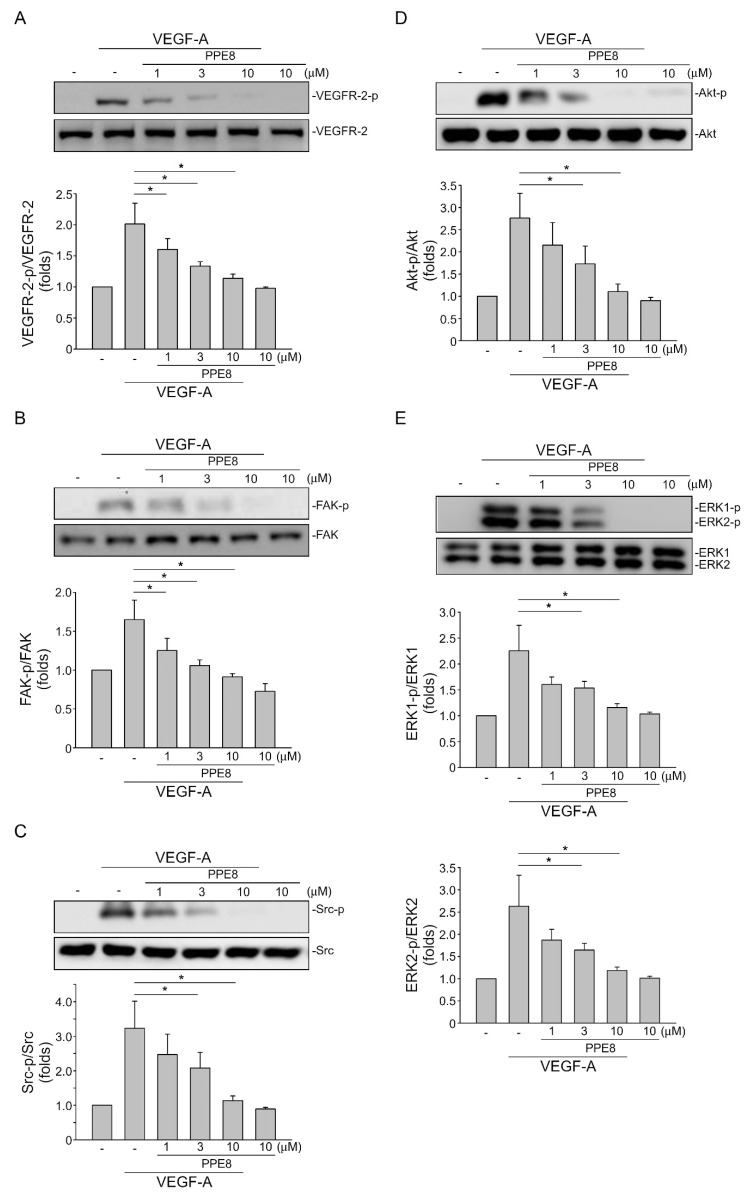
PPE8 inhibited endothelial VEGF-A–VEGFR-2 signaling. After 30 min treatment of PPE8, HUVECs were stimulated with VEGF-A (25 ng/mL) for another 5 (VEGFR-2) or 30 (ERK1/2, Akt, FAK, and Src) min. The status of VEGFR-2 Tyr1175 (**A**), FAK Tyr397 (**B**), Src Tyr416 (**C**), Akt Ser473 (**D**), or ERK1/2 Thr202/Tyr204 (**E**) phosphorylation was examined by immunoblotting. The compiled results are shown in the bottom of the chart. Each column represents the mean ± S.E.M. of five independent experiments. * *p* < 0.05, compared with the group treated with VEGF-A alone; ANOVA on ranks, with Kruskal–Wallis test.

VEGF-A–VEGFR-2 signaling plays a key role in modulating tissue vascularization and blood vessel homeostasis, as well as vascular-related diseases [35]. VEGFR-2 becomes phosphorylated on several tyrosine residues upon VEGF-A exposure. It is believed that VEGFR-2 Tyr 1175 phosphorylation, among these residues, is crucial in triggering downstream signaling cascades including ERK1/2, Akt, Src, and focal adhesion kinase (FAK), to induce angiogenesis [36]. Therefore, we sought to examine whether PPE8 affects VEGFR-2 Tyr 1175 phosphorylation in VEGF-A-stimulated HUVECs. As shown in Figure 4, PPE8 significantly reduced VEGFR-2 Tyr 1175 phosphorylation after VEGF-A exposure (Figure 4A). PPE8 also inhibited VEGF-A-induced phosphorylation of FAK (Figure 4B), Src (Figure 4C), Akt (Figure 4D), and ERK1/2 (Figure 4E). Together these observations indicate that PPE8 may exhibit anti-angiogenic activities via targeting endothelial VEGF-A–VEGFR-2 signaling.

### 3.5. PPE8 Attenuated Melanoma Growth In Vivo 

**Figure 5:**
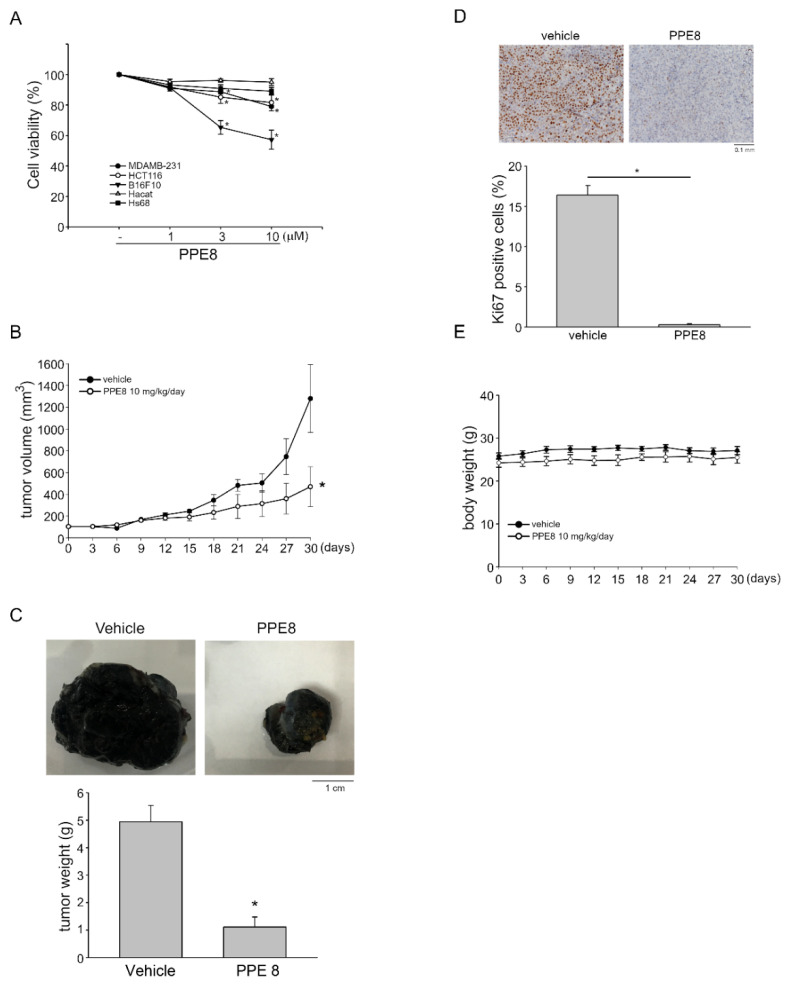
PPE8 suppressed B16F10 melanoma xenograft growth in vivo. (**A**) MDA-MB-231, HCT116, B16F10, Hacat, or Hs68 cells were treated with indicated concentrations of PPE8 for 24 h. MTT assay was employed to determine cell viability. Values represent the mean ± S.E.M. of six independent experiments performed in duplicate. * *p* < 0.05, compared with the control group (**B**) C57BL/6 mice bearing B16F10 melanoma xenografts were intraperitoneally administrated with vehicle or PPE8 (10 mg/kg/day) for 30 days. Tumor volume was evaluated as described in the “Materials and Methods” section. Values represent the mean ± S.E.M. of six xenografts. (**C**) Mice were sacrificed at the end of the experiment (30-days treatment) and tumors were harvested and weighed. Each column represents the mean ± SEM of six xenografts; * *p* < 0.05, compared with the vehicle-treated control group; Student’s *t*-test (**D**) Cryosections of B16F10 xenografts were stained with anti-Ki67 antibody for detecting proliferative cells. Immunohistochemical images shown are representative of six independent experiments with similar results. Each column represents the mean ± SEM of six independent experiments; * *p* < 0.05, compared with the vehicle-treated control group; Student’s *t*-test (**E**) We examined body weights of the C57BL/6 mice every 3 days within 30 days of treatment. Values represent the mean ± SEM of six mice.

PPE8 not only exhibit anti-angiogenic actions but may also possess anti-tumor activities. Results derived from the MTT assay demonstrate that PPE8 did not alter cell viability in non-tumor HaCat keratinocytes and Hs68 fibroblasts (Figure 5A). However, PPE8 at 10 μM caused approximately a 20% reduction in cell viability in both HCT116 colorectal cancer cells and MDA-MB-231 breast cancer cells (Figure 5A). In contrast, PPE8 had a more obvious effect on the inhibition of cell viability in B16F10 melanoma cells (Figure 5A). We used a xenograft murine model to further investigate whether PPE8 exhibits anti-melanoma activities in vivo. B16F10 melanoma cells were implanted and allowed to grow to an average size of approximately 100 mm^3^. Mice were intraperitoneally administered with vehicle or PPE8 (10 mg/kg/day) for 30 days and xenografts were harvested at the end of the treatment. As shown in Figure 5B, PPE8 markedly suppressed B16F10 tumor xenograft growth as compared to the vehicle-treated control group. Tumor weight was also reduced in the presence of PPE8 (Figure 5C). We examined whether PPE8 affects cell proliferation in B16F10 melanoma xenografts using immunohistochemistry with Ki-67 staining, a specific marker of the proliferating cell. As shown in Figure 5D, PPE8 significantly reduced the number of Ki-67-positive cells, indicative of reduced cell proliferation. Moreover, compared to the vehicle-treated control group, systemic administration of PPE8 (10 mg/kg/day) was without effect on mouse body weight within 30 days (Figure 5E). These findings indicate that PPE8 at 10 mg/kg/day is capable of reducing tumor growth in vivo.

## 4. Discussion

Cancer is the leading cause of death globally and has a significant impact on healthcare and society. Although the number of cancer survivors has increased due to the advances in therapeutic modalities in developed countries, continuous development of therapeutic strategies or agents that effectively attenuate tumor progression remains critical. It is believed that cancers require neovascularization to grow and metastasize, which accounts for approximately 90% of cancer-related deaths. Targeting tumor vasculature not only prevents tumors from growing and metastasizing [37] but also improves tumor responses to chemotherapy [38,39] and enhances host anti-tumor immunity [3,7,40]. Angiogenesis inhibition is thus regarded as essential to normalize tumor-associated vasculature and serves as an adjuvant strategy in the treatment of cancer [40,41,42].

Naturally occurring quinones and their derivatives have been recognized for numerous years as a vital source of novel therapeutic agents. Based on the aromatic structural pattern, quinones have been classified into three classes: benzoquinonoes, naphthquinones, and anthraquinones [43]. Of these, naphthoquinones have recently attracted considerable attention for their potential as anti-cancer agents [26,27]. We recently identified a novel 1,4 naphthoquinone-based compound, PPE8, that exhibits antitumor effects in non-small cell lung cancer cells [29]. In the present study, we further demonstrated that *PPE8* may exhibit anti-angiogenic properties via suppressing VEGF-A–VEGFR-2 signaling. Moreover, *PPE8* also significantly reduced melanoma growth in a murine xenograft model.

1,4-Naphthoquinone has been recognized as an angiogenesis inhibitor [44]. However, a recent study showed that 1,4-naphthoquinone itself may not be used as an anti-angiogenic agent since it exhibits cytotoxic effects on endothelial cells and fibroblasts [30]. Several 1,4-naphthoquinone derivatives such as plumbagin [45] and 6-TMNQ [30] exhibit anti-angiogenic properties. However, their anti-angiogenic actions may involve VEGFR-2-independent signaling. We noted in this study that PPE8 is without effects on non-cancerous cell viability and is likely safer in posing a lower risk of bleeding. Together with the observations that systemic administration of PPE8 did not alter the body weight of the mice, this suggests that PPE8 may represent an effective anti-angiogenic candidate via targeting VEGF-A–VEGFR-2 signaling with less toxicity.

Although angiogenesis involves intertwined signaling pathways, targeting VEGF-A–VEGFR-2 signaling, the most critical regulator of angiogenesis, remains the major focus in the oncology field to discover novel anti-angiogenic agents [46,47]. Upon VEGF-A binding, VEGFR-2 undergoes dimerization and phosphorylation on certain tyrosine residues, in particular, Tyr 1175 (Y1175), to activate downstream signaling cascades such as ERK1/2, Akt, Src, or FAK that promotes angiogenesis [12,13]. Zhu et al. [48] recently showed the beneficial effects of inhibition of ERK1/2, Akt, Src, or FAK signaling downstream of VEGFR-2 for breast cancer treatment. In agreement with these results, we show that PPE8 reduces VEGF-A-induced phosphorylation of VEGFR-2 Tyr 1175, FAK, Src, Akt, and ERK1/2 in HUVECs. In in vivo models, PPE8 also suppressed VEGF-A- or breast-cancer-cell-induced angiogenesis. Although VEGFR-2 signaling is crucial for angiogenesis, other RTK signaling pathways such as FGFR [49] and PDGFR [50] also contribute to angiogenesis. The crosstalk between VEGF-A and bFGF signaling has been reported in neovascularization [51]. Murota et al. [30] recently showed that 6-TMNQ, a novel 1, 4 naphthoquinone derivative, exhibits anti-angiogenic properties without affecting endothelial VEGF-A–VEGFR-2 signaling. This raises the possibility that more than one pro-angiogenic factor may co-operate with VEGF-A in neovascularization. Whether PPE8 exhibits inhibitory actions against these angiogenic RTK pathways remains to be investigated. Taken together, PPE8 may inhibit angiogenesis through, at least in part, interfering with the VEGF-A–VEGFR-2 signaling pathway. 

The underlying mechanisms of PPE8 in reducing VEGF-A–VEGFR-2 signaling and angiogenesis remain incompletely understood. Several novel naphthoquinone derivatives have been shown to exhibit pharmacological properties via antagonizing P2x 7 purinergic receptor [52] or G-protein-coupled receptor 55 (GRP55) [53]. Munni et al. [54] recently demonstrated that shikonin with naphthoquinone phamacophore may bind to the catalytic domain of VEGFR-2 using molecular docking simulations. In addition, VEGFR-2 phosphorylation is also regulated by protein tyrosine phosphatases such as SHP-1 [55,56], VE-PTP [57], or density enhanced phosphatase (DEP)-1 [55]. It raises the possibility that PPE8 may interact with VEGFR-2 or VEGF-A to antagonize VEGF-A–VEGFR-2 signaling. PPE8’s anti-angiogenic actions may also involve the modulation of protein phosphatases. Further investigations are needed to characterize the precise mechanisms by which PPE8 suppresses VEGF-A–VEGFR-2 signaling.

Besides anti-angiogenic actions, PPE8 likely has additional anti-tumor effects. We noted that PPE8 did not alter cell viability in non-tumor Hs68 fibroblasts or HaCat keratinocytes. However, PPE8 reduced cell viability with an approximately 20% reduction in both HCT 116 colorectal cancer and MDA-MB-231 breast cancer cells. In contrast, PPE8 caused a noticeable reduction in B16F10 melanoma cell viability. PPE8 also significantly reduced melanoma growth in vivo. It is likely that PPE8 is more effective in reducing cell viability in murine B16F10 melanoma cells than in human cancer cell lines. Whether PPE8 is also effective against human melanoma cells needs to be further investigated. It appears that PPE8’s actions in decreasing tumor cell viability may vary among different histological origins or different types of cancer. The underlying mechanisms of these differences remain unclear. It has been thought that different cancer types may exhibit differential sensitivities to chemotherapeutic agents due to their different doubling times [58]. In addition, the difference of PPE8’s cytotoxic effects in these cancer cell lines may be due to different cellular contents in each cancer type.

Tas et al. [59] demonstrated that high serum VEGF levels associate with tumor progression and poor prognosis in patients with melanoma. VEGFRs could also be detected in various types of human tumor cells including melanoma cells, although VEGFRs are regarded as endothelial receptors [60,61]. However, the functionality of VEGFs or VEGFRs expressed in tumor cells remains unclear. It appears that VEGF may activate autocrine or paracrine loops to stimulate tumor cell proliferation and invasion [61,62,63,64,65]. Whether PPE8 exhibits anti-tumor effects against other types of cancer and the causal role of VEGF–VEGFR signaling remain to be delineated. It is also worth clarifying whether VEGFR-independent signaling contributes to PPE8’s anti-tumor actions. 

In conclusion, we show in the present study that PPE8, a novel 1, 4 naphthoquinone derivative, exhibits anti-angiogenic activities through suppressing endothelial VEGF-A–VEGFR-2 signaling. PPE8 may also have additional anti-tumor properties. Although the mechanisms underlying these actions remain to be established, these findings support the role of PPE8 as a potential drug candidate for the development of therapeutic agents in the treatment of angiogenesis-related diseases including cancer.

## Data Availability

Not applicable.

## Supplementary Materials

The following are available online at https://www.mdpi.com/article/10.3390/cells11132114/s1, Figure S1: Effects of PPE and PPE8 on VEGF-A-induced cell proliferation in HUVECs, Figure S2: Effects of PPE8 on tail bleeding times of mice.
