# Supplementary material for: Suppressing VEGF-A/VEGFR-2 Signaling Contributes to the Anti-Angiogenic Effects of PPE8, a Novel Naphthoquinone-Based Compound
Source: Cells. 2022 Jul 5;11(13):2114. doi: 10.3390/cells11132114 (PMC9266117; doi:10.3390/cells11132114)
Supplement: Supplementary file 1 [file cells-11-02114-s001.zip › cells-1785697-supplementary.pdf]

Supplement Information

Supplement Methods

Tail bleeding time analysis

Male C57BL/6 mice (4-week-old) purchased from the National Laboratory Animal Center (Taipei, Taiwan) were employed to examine the effects of PPE8 on tail bleeding time. Animals were randomized to either the PPE8-treated group or vehicle-treated control group. Mice were intraperitoneally administrated with PPE8 (10 mg/kg/day) once daily for 10 days. At the end of treatment, the mouse was placed in a tube holder with its tail protruding and a 2 mm segment from the distal tail was severed. The amputated tail was immediately immersed in isotonic saline at 37℃. Bleeding time was recorded for a maximum of 1800 s and the endpoint was the arrest of bleeding (Lien et al., 2019).

Supplement Figures

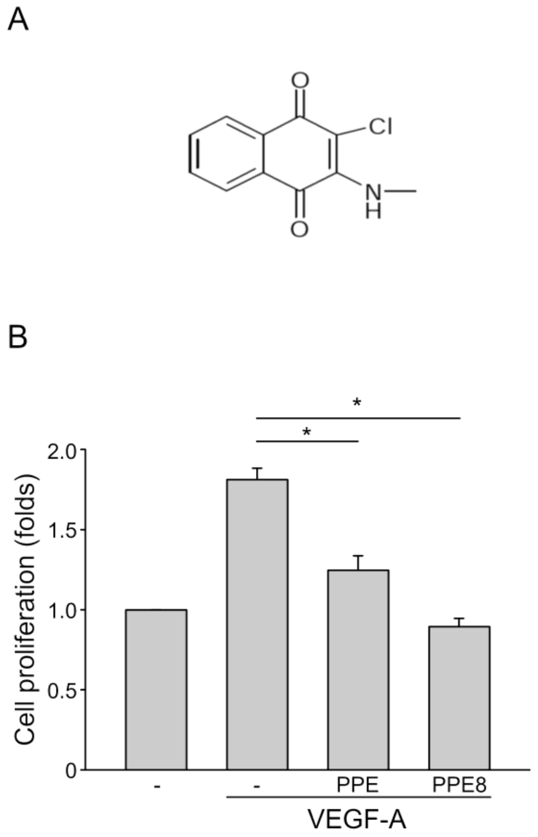

Supplement Figure S1. Effects of PPE and PPE8 on VEGF-A-induced cell proliferation in HUVECs (A) Chemical structures of PPE. (B) HUVECs were starved in 2 % FBS-containing M199 without ECGS for 18 h. After starvation, cells were treated with PPE or PPE8 at 10 μM, followed by the stimulation with VEGF-A (25 ng/ml) for another 24 h. A BrdU-based cell proliferation assay was used to determine cell proliferation. Each column represents the mean ± S.E.M. of five independent experiments performed in duplicate. *P < 0.05, compared with the group treated with VEGF-A alone; Kruskal-Wallis test.

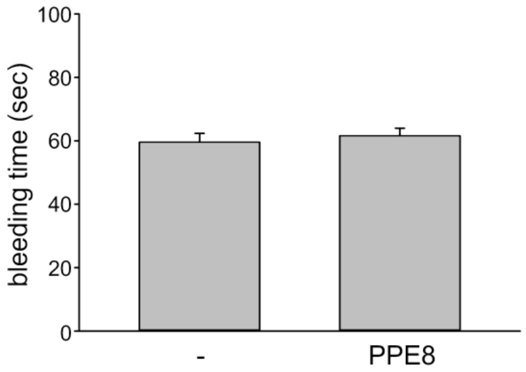

Supplement Figure S2. Effects of PPE8 on tail bleeding times of mice. Mice were intraperitoneally administrated with vehicle or PPE8 (10 mg/kg/day) for 10 days. Tail bleeding time was determined. Each column represents the mean ± S.E.M. (N=5 for each group). Sec: second
